# Randomized Controlled Trial of Simulation vs. Standard Training for Teaching Medical Students High-quality Cardiopulmonary Resuscitation

**DOI:** 10.5811/westjem.2018.11.39040

**Published:** 2018-12-12

**Authors:** C. Eric McCoy, Asif Rahman, Juan C. Rendon, Craig L. Anderson, Mark I. Langdorf, Shahram Lotfipour, Bharath Chakravarthy

**Affiliations:** University of California Irvine School of Medicine, Department of Emergency Medicine, Irvine, California

## Abstract

**Introduction:**

Most medical schools teach cardiopulmonary resuscitation (CPR) during the final year in course curriculum to prepare students to manage the first minutes of clinical emergencies. Little is known regarding the optimal method of instruction for this critical skill. Simulation has been shown in similar settings to enhance performance and knowledge. We evaluated the comparative effectiveness of high-fidelity simulation training vs. standard manikin training for teaching medical students the American Heart Association (AHA) guidelines for high-quality CPR.

**Methods:**

This was a prospective, randomized, parallel-arm study of 70 fourth-year medical students to either simulation (SIM) or standard training (STD) over an eight-month period. SIM group learned the AHA guidelines for high-quality CPR via an hour session that included a PowerPoint lecture with training on a high-fidelity simulator. STD group learned identical content using a low-fidelity Resusci Anne® CPR manikin. All students managed a simulated cardiac arrest scenario with primary outcome based on the AHA guidelines definition of high-quality CPR (specifies metrics for compression rate, depth, recoil, and compression fraction). Secondary outcome was time to emergency medical services (EMS) activation. We analyzed data via Kruskal-Wallis rank sum test. Outcomes were performed on a simulated cardiac arrest case adapted from the AHA Advanced Cardiac Life Support (ACLS) SimMan® Scenario manual.

**Results:**

Students in the SIM group performed CPR that more closely adhered to the AHA guidelines of compression depth and compression fraction. Mean compression depth was 4.57 centimeters (cm) (95% confidence interval [CI] [4.30–4.82]) for SIM and 3.89 cm (95% CI [3.50–4.27]) for STD, p=0.02. Mean compression fraction was 0.724 (95% CI [0.699–0.751]) for SIM group and 0.679 (95% CI [0.655–0.702]) for STD, p=0.01. There was no difference for compression rate or recoil between groups. Time to EMS activation was 24.7 seconds (s) (95% CI [15.7–40.8]) for SIM group and 79.5 s (95% CI [44.8–119.6]) for STD group, p=0.007.

**Conclusion:**

High-fidelity simulation training is superior to low-fidelity CPR manikin training for teaching fourth-year medical students implementation of high-quality CPR for chest compression depth and compression fraction.

## INTRODUCTION

Cardiovascular disease (CVD) accounts for 33.6% of all-cause mortality, or one of every three deaths in the United States (U.S.) annually. On average, more than 2,200 Americans die of CVD each day, approximately one death every 39 seconds.^1^ CVD claims more lives each year than cancer, chronic lower respiratory disease, and accidents combined.^2^

The impact of out-of-hospital cardiac arrest (OHCA) is substantial, claiming nearly 300,000 lives annually.^1^ Although survival rates vary widely, they are still generally low (<10%) in most areas of the country.^3^ However, many communities have significantly improved survival rates. The focus in communities saving the most lives from OHCA has been high-quality cardiopulmonary resuscitation (CPR). Growing evidence suggests that simple changes in CPR technique, with emphasis on ensuring proper compression rate, depth, chest wall recoil, minimizing interruptions and avoiding over-ventilation, markedly improve survival.^4^–^6^

Although the concepts of CPR are becoming better understood, there remains a large chasm between what we know and how it is performed on patients, in both out-of-hospital and in-hospital settings. Despite the fact that CPR is a critical link in the chain of survival, it is performed with inconsistent quality in both settings.^7^–^8^ The American Heart Association (AHA) CPR Guidelines emphasize that, to close the knowledge-practice gap and save more lives, providers should develop a culture of measuring and ensuring high-quality CPR.^9^–^10^

Human patient simulation provides the opportunity to address the knowledge-practice gap in the education, training, and implementation of high-quality CPR. Simulation encompasses any technology or process that re-creates a contextual background that allows a learner to experience success, mistakes, receive feedback, and gain confidence in a learner-oriented environment void of patient risk.^11^ The Institute of Medicine, the Educational Technology Section of the Academic Emergency Medicine Consensus Conference, and the public have advocated for increased simulation training to reduce medical error.^12^–^16^ Basic life support (BLS) and Advanced Cardiac Life Support (ACLS) have been recognized as the standard criteria for competency to manage patients in cardiac arrest. Written evaluation is not a predictor for skills performance in an ACLS course, and there is a paucity of randomized studies comparing the effectiveness of simulation vs. standard teaching/training in retention of ACLS knowledge, as well as ability to manage critically ill patients.^17^–^19^ Our study compares the effectiveness of high-fidelity simulation vs. traditional low-fidelity manikin training for medical students in the AHA BLS CPR guidelines for chest compression rate, depth, recoil, and compression fraction.

## METHODS

### Study Design and Setting

We conducted this prospective, randomized, parallel-group study in a simulation center at a University of California (UC) medical school over an eight-month period. The UC Irvine Health Medical Education Simulation Center is a 65,000-square foot, state-of-the-art facility that provides telemedicine and simulation-based educational programs and continuing medical education courses for thousands of healthcare providers each year.^20^ Resources for education and training include a full-scale operating room, emergency department (ED) trauma bay, obstetrics suite, and a critical care unit. The simulation center has a complement of full-time staff, including full-time simulation specialists.

**Population Health Research Capsule**

What do we already know about this issue?

*Although cardiopulmonary resuscitation (CPR) is considered the most vital element in the chain of survival for cardiac arrest, little is known regarding the optimal method of instruction for this critical skill*.

What was the research question?

*We evaluated the comparative effectiveness of high- vs. low-fidelity simulation training for teaching high-quality CPR*.

What was the major finding of the study?

*Students trained with high-fidelity simulation performed CPR that more closely adhered to the American Heart Association CPR guidelines*.

How does this improve population health?

*Optimal CPR education and training for healthcare providers at the curriculum level allows the opportunity to optimize the performance of this critical skill at the population level*.

### Selection of Participants

All fourth-year medical students enrolled in a required emergency medicine (EM) clerkship were eligible. We excluded foreign medical students doing an observation rotation in the ED to evaluate a representative group of U.S. medical students. The EM clerkship includes a simulation component. During clerkship orientation each month, students were offered voluntary participation in the study. Use of the simulator was not restricted to the study, and results of the study did not affect clerkship evaluation. The study was approved by the university’s institutional review board, and subjects provided informed consent.

### Interventions

Participants were randomized to control or intervention groups with a computerized, random-number generator using block sizes of four. After randomization, all students received an equivalent orientation to the human patient simulator (Laerdal SimMan® 3G full-scale patient simulator [Laerdal Medical Corporation, Wappingers Falls, New York]), which included introducing and reviewing simulator features as well as the physiologic monitoring devices available. Students were instructed to verbalize their thoughts, orders, and actions during the simulated patient scenario. The students were unaware of the simulation case they would manage. All participants had previous experience with the simulator.

Both groups received a didactic lecture via PowerPoint (Microsoft Corporation, Redmond, Washington) on the AHA Guidelines for CPR and Emergency Cardiovascular Care (ECC).^21^ The Guidelines for CPR and ECC are based on the International Liaison Committee on Resuscitation (ILCOR) International Consensus on CPR and ECC Science with Treatment Recommendations.^22^,^23^

The practical skills component took place directly after didactics. This session consisted of training the medical students to perform high-quality CPR as specifically defined and highlighted in the ILCOR guidelines.^23^ The components of high-quality CPR pertain to chest compression rate, depth, recoil, and compression fraction. The students practiced CPR with a specific focus on these four components. The education and training during this session was the same between the intervention and control groups, with the exception of the types of manikins used (high-fidelity vs. low-fidelity). The high-fidelity manikin provided real-time feedback during student CPR on chest compression rate, depth, and recoil. The low-fidelity manikin does not provide such real-time feedback.

Feedback to the participants in the control group was given after they performed CPR. This type of post-performance feedback is similar to that given in all CPR training courses in North America where low-fidelity manikins (that do not provide real-time feedback) are used. The instruction during the practical skills component of the course was identical for the intervention and control groups, with the only exception being the type of manikin the students were randomized to. The intervention group received their training on the high-fidelity human patient simulator, and the control group received their training on the standard, low-fidelity CPR procedural tasks trainer Resusci Anne® (Laerdal Medical Corporation, Wappingers Falls, New York).

### Methods and Measurements

The performance metrics measured for high-quality CPR in our study were specifically defined in the AHA Guidelines for CPR and ECC and included chest compression rate, depth, recoil, and compression fraction. The high-fidelity simulation software allows real-time collection of chest compression rate, depth, and recoil data. The precision of compression rate is to the nearest full compression, depth to the nearest millimeter, and recoil to the nearest percent (100% release recoil indicating all compressions delivered during a cycle were accompanied by adequate chest recoil). Video capture of each scenario was performed with B-line Medical SimBridge® software (B-line Medical, Washington, District of Columbia).

We defined performance metrics prior to study implementation. Compression rate was defined as the number of chest compressions delivered per minute. Compression depth was defined as depth of chest compression from neutral position of the sternum in centimeters. We defined chest recoil as allowing the sternum to fully (100%) return to its neutral position before the next chest compression. Compression fraction was defined as the proportion of time CPR was delivered while the patient was without a perfusing rhythm. The total time measured for absence of a perfusing rhythm began with the initiation of ventricular fibrillation and ended with the completion of the tenth cycle of CPR. For those subjects who chose the hands-only CPR methods, the end time was marked when they delivered 300 compressions. This allowed measurement of their performance after the same 300 compressions delivered in the 10-cycle CPR group. The time to emergency medical services (EMS) activation was defined as the time from ventricular fibrillation onset to when the participant verbalized request to activate EMS. Activating emergency response is the first step in the AHA adult cardiac arrest algorithm.

The simulation case used was that of an elderly male suffering a cardiac arrest, which was adapted from the AHA ACLS SimMan® Scenarios set. Human resources used for the evaluation scenarios consisted of a full-time simulation specialist, a researcher to oversee correct implementation of the study protocol, and a confederate in the scenario to provide ancillary support.

Data input was done via standardized data abstraction sheets. Data abstractors were trained through an instructional workshop detailing definitions of the performance metrics as well as how to input data into collection sheets. We implemented double data entry to minimize random data abstraction errors. Discrepancies were resolved by reviewing original data in the recordings to check abstraction accuracy. We input all data into a master data spreadsheet file.

### Outcomes

The AHA Guidelines for CPR and ECC are based on the ILCOR International Consensus on CPR and ECC Science with Treatment Recommendations.^22^,^23^ High quality for rate was defined as ≥100 compressions/minute, depth ≥5 centimeters (cm), allowing full (100%) chest recoil, and a compression fraction that approached 100%. The guidelines also state that for the treatment of cardiac arrest, ACLS interventions build on the BLS foundation of immediate recognition and activation of EMS.^21^ This was the driver behind our secondary outcome of time to activation of EMS, defined as the time from cardiac arrest (ventricular fibrillation) to the time the student verbalized a request to activate EMS. All outcome data were obtained during a high-fidelity cardiac arrest simulation scenario adapted from the AHA ACLS SimMan® Scenario set.

### Analysis

Data from the master data collection sheet were converted to Stata file format and analyzed with Stata (version 12.0; StataCorp, College Station, Texas). We reported continuous variables as means with 95% confidence intervals (CI) using the Kruskal-Wallis rank sum test. A two-tailed alpha <0.05 represented statistical significance. Our sample size calculations were based on an effect size (difference in means) of a 5 millimeter (mm) difference in compression depth between the two groups. With a two-tailed alpha (α) of <0.05, and a beta (β) of 0.2, we needed 34 subjects per group to detect a difference between groups with a power of 0.8.

## RESULTS

Of 74 eligible participants, data on 70 were available for analysis as four participants were absent for their assigned simulation session (Figure). For our primary outcome, the mean compression depth was 4.57 cm (95% CI [4.30 – 4.82]) for the SIM group and 3.89 cm (95% CI [3.50 – 4.27]) for the standard (STD) group, p=0.02. The compression fraction was 0.724 (95% CI [0.699 – 0.751]) for the SIM group and 0.679 (95% CI [0.655 – 0.702]) for the STD group, p=0.01. The mean compression rate was 123.3 per minute (95% CI [117.9 – 128.4]) for the simulation (SIM) group and 116.1 per minute (95% CI [109.9 – 121.2]) for the STD group, p=0.06. The mean percentage of chest compressions that were accompanied by full chest recoil was 0.954 (95% CI [0.925 – 0.978]) for the SIM group and 0.941 (95% CI [0.874 – 0.985]) for the STD group, p=0.83 (Table).

For our secondary outcome, the time to activation of EMS was 24.7 seconds (95% CI [15.7 – 40.8]) for the SIM group and 79.5 seconds (95% CI [44.8 – 119.6]) for the STD group, p=0.007 (Table).

## DISCUSSION

In our prospective, randomized, parallel-group study evaluating the comparative effectiveness of high-fidelity simulation training vs. standard training, we found that high-fidelity simulation training yielded CPR performance that more closely adhered to the AHA CPR guidelines. To our knowledge, this is the first report documenting improved performance of medical students with high-fidelity simulation to teach high-quality CPR. Specifically, we observed superior performance of chest compression depth and compression fraction, metrics explicitly stated by the AHA to be components of high-quality CPR. We also observed a more rapid activation of the EMS system by the simulation-trained group. The AHA’s recommendation and emphasis on these metrics are supported by recent studies that have demonstrated improved outcomes from OHCA and have reaffirmed the importance of a stronger emphasis on adequate compression rate, depth, recoil, and compression fraction.^24^–^31^ Conversely, our training innovation had no measured effect on compression rate or recoil.

A few points of emphasis on major recommendations in the guidelines have particular relevance for simulation. First is the recommendation that “manikins with realistic features such as the capability to replicate chest expansion and breath sounds, generate a pulse and blood pressure, and speak may be useful for integrating the knowledge, skills, and behaviors required in ALS training.”^32^ Second is that “written tests should not be used exclusively to assess the competence of a participant in an advanced life support course,” as there needs to be a performance assessment as well. Third, “CPR prompt and feedback devices may be useful for training rescuers and may be useful as part of an overall strategy to improve the quality of CPR for actual cardiac arrest.”^32^

Our findings that simulation yields student performance more closely adherent to AHA guidelines are consistent with a growing body of literature supporting simulation in resuscitation research and training. Research integrating high-fidelity simulation with ACLS training has found that a simulation-based ACLS course significantly improved knowledge, psychomotor skills, and performance during resuscitation.^33^ A prospective, randomized study across 10 institutions running a standardized simulated cardiopulmonary arrest scenario concluded that using novel and practical technology can improve compliance with the AHA guidelines for CPR that are associated with better outcomes.^34^ There has also been simulation-based research showing that real-time resuscitation guidance significantly increases adherence to the AHA guidelines.^35^ The use of high-fidelity simulation has also shown benefit in CPR knowledge, skills, acquisition, retention, and advanced resuscitation in the disciplines of nursing and pharmacy.^36^,^37^ A recent systematic review and meta-analysis evaluating simulation technology for resuscitation training concluded that simulation-based training for resuscitation is highly effective.^38^

Our study contributes to the simulation literature that advances scientific knowledge in the area of simulation education, provides guidance for future areas of research, and also offers insight for those stakeholders who play a significant role in the creation of policies, protocols or procedures in the practice of simulation-based education. Our study adds to the body of simulation literature in a number of ways. The majority of interventional studies in simulation-based training use non-experimental study designs (i.e., non-randomized study designs) to evaluate the effect of simulation. Our study consisted of a prospective, randomized controlled trial study design, which carries less risk of bias when compared to non-randomized study designs. Randomization allows the differences in outcome of a study to be attributed to the intervention with more confidence than any other study design. Our study is also unique in that we used performance metrics of high-quality CPR specifically defined by the AHA guidelines as the primary outcome, whereas previous studies did not measure all the performance metrics or found no significant difference in outcomes.^34^,^35^

There is also literature that has shown no benefit to simulation training in resuscitation. A prospective study evaluating whether simulation-based ACLS training improves performance in managing simulated and actual cardiac arrest found no difference in adherence to the AHA guidelines.^39^ Another study evaluated whether participants who receive ACLS training on high-fidelity manikins performed better than those trained on low-fidelity manikins found no difference in groups on written tests scores.^40^ Some of the literature pertaining to simulation in resuscitation care has limitations including selection bias, heterogeneity of outcome measures, study design lacking robust methodologies, and small samples leading to underpowered studies unable to detect a true difference between groups.

For our secondary outcome, we observed a more rapid activation of the EMS system by the simulation-trained group, by an average of 55 seconds. Activating emergency response is the first step in the adult cardiac arrest algorithm. Research has shown that for victims of witnessed ventricular fibrillation arrest, early CPR and rapid defibrillation can significantly increase chance of survival to hospital discharge.^41^–^46^ Implementing education and training strategies designed to measure and improve these metrics has the potential to maximize patient outcomes.

We believe that feedback in high-fidelity simulation is a key driver behind performance enhancement as students get to actually experience what the correct compression rate, depth, recoil and compression fraction feel like. The real-time feedback allows the learner to make immediate adjustments to their performance and gain confidence that their actions yield the desired result(s). We believe the feedback that is provided through high-fidelity simulation is what resulted in superior CPR performance in the SIM group. We believe the deeper a learner can be immersed in a training environment, the more closely their actions will reflect what they have learned and practiced.

In training, the SIM group received feedback from the simulator indicating the chest compression rate and depth. We observed what appeared to be fatigue at a faster rate in those students performing at adequate rate and depth and also noted they were quicker to call for help (EMS activation). This observation is quantified in the difference in time to EMS activation between the two groups. We believe a combination of participant fatigue during CPR performance assessment and the fidelity of immersion during the practical skills training are two variables that contributed to this difference. Research has shown that having the knowledge of CPR is necessary but not sufficient to actually perform with high adherence to the AHA guidelines.^47^ Simulation-based training allows the quantitative measurement of performance during CPR and provides a means to measure improvement.

## LIMITATIONS

Our study did not evaluate the educational intervention on actual cardiac arrest patients. It may have suffered as well from simulator bias, as the STD training group had less experience with the high-fidelity simulator prior to testing in high fidelity. However, all the participants had high-fidelity simulation incorporated into their core medical school curriculum and were familiar with the simulator at study onset. This experience and familiarity with the high-fidelity simulator strongly argue against any significant potential impact of simulator bias. Furthermore, all students were oriented to the high-fidelity simulator as part of the study protocol to standardize their experience and familiarity with the manikin.

Our primary outcome of high-quality CPR was composed of four performance metrics. Increasing the number of outcome measures increases the potential for a type I error. The AHA emphasizes that high-quality CPR has multiple components, and we felt it important to address each of these metrics independently instead of creating a summary metric.

Instructors were not blinded to the educational modality they were using to teach students as there are readily apparent differences between the high- and low-fidelity simulators. We did not perform a longitudinal study and therefore cannot comment on the long-term benefit of this type of intervention. And finally, we did not find a statistically significant difference between compression rates. Compression rates in both groups were in compliance with the AHA CPR recommendations, so there was no opportunity to find improvement.

## CONCLUSION

In our prospective, randomized, parallel-group study evaluating the comparative effectiveness of high-fidelity simulation training vs. standard training, we found that high-fidelity simulation training yielded CPR performance that more closely adheres to AHA CPR guidelines. Simulation-trained participants also had shorter times to EMS activation, the first step in the AHA adult cardiac arrest algorithm. Further research is needed to evaluate the most effective teaching methods for cardiac arrest care.

## Figures and Tables

**Figure:**
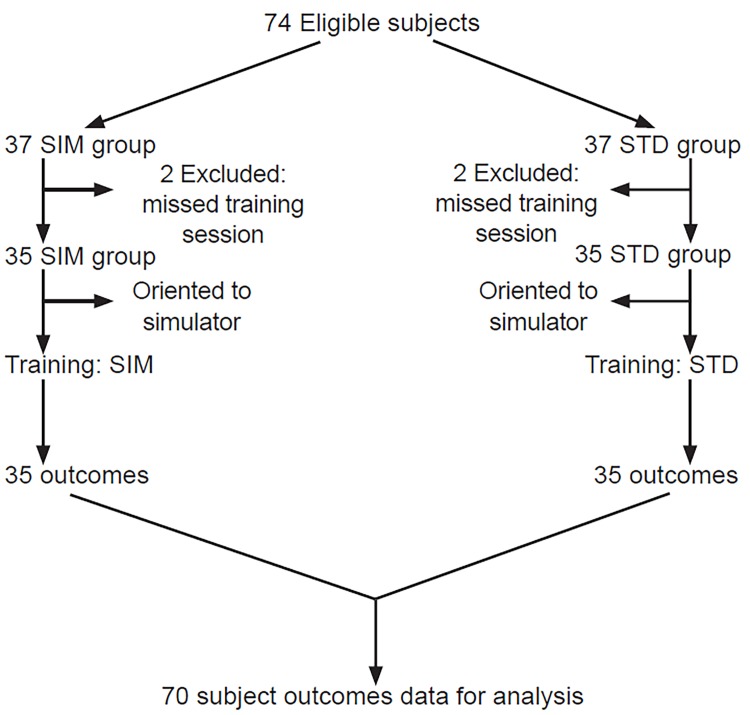
Flow sheet of participants in study comparing high-fidelity simulation to standard training. *SIM*, high-fidelity simulation; *STD*, standard training with low-fidelity Resusci Anne®.

**Table:** Main outcome variables according to teaching method.

Variable	Teaching method	Mean	95% CI	P value
Compression rate/min	STD	116.1	109.9–121.2	.06
	SIM	123.3	117.9–128.4	
Depth (cm)	STD	3.89	3.50–4.27	.02
	SIM	4.57	4.30–4.82	
Recoil proportion	STD	.941	.874–.985	.83
	SIM	.954	.925–.978	
Compression fraction	STD	.679	.655–.702	.01
	SIM	.724	.699–.751	
Time to EMS activation (seconds)	STD	79.5	44.8–119.6	.007
	SIM	24.7	15.7–40.8	

*SIM*, simulation training group; *STD*, standard training group; *CI*, confidence interval; *cm*, centimeter; *EMS*, emergency medical services; *min*, minute.

Compression rate/min = number of chest compressions delivered per minute; recoil proportion = proportion of compressions accompanied by 100% chest recoil; compression fraction = proportion of time compressions performed while patient in a non-perfusing rhythm.
